# Reply to Ayuso García et al. Health Perception among Female COVID-19 Patients. Comment on “Fernández-de-las-Peñas et al. Female Sex Is a Risk Factor Associated with Long-Term Post-COVID Related-Symptoms but Not with COVID-19 Symptoms: The LONG-COVID-EXP-CM Multicenter Study. *J. Clin. Med.* 2022, *11*, 413”

**DOI:** 10.3390/jcm11133616

**Published:** 2022-06-23

**Authors:** César Fernández-de-las-Peñas, José D. Martín-Guerrero, Óscar J. Pellicer-Valero, Esperanza Navarro-Pardo, Víctor Gómez-Mayordomo, María L. Cuadrado, José A. Arias-Navalón, Margarita Cigarán-Méndez, Valentín Hernández-Barrera, Lars Arendt-Nielsen

**Affiliations:** 1 Department of Physical Therapy, Occupational Therapy, Physical Medicine and Rehabilitation, Universidad Rey Juan Carlos (URJC), 28922 Madrid, Spain; 2 CNAP, Center for Sensory-Motor Interaction (SMI), Department of Health Science and Technology, Faculty of Medicine, Aalborg University, 9220 Aalborg, Denmark; lan@hst.aau.dk; 3 Intelligent Data Analysis Laboratory, Department of Electronic Engineering, ETSE (Engineering School), Universitat de València (UV), 46100 Valencia, Spain; jose.d.martin@uv.es (J.D.M.-G.); oscar.pellicer@uv.es (Ó.J.P.-V.); 4 Department of Developmental and Educational Psychology, Universitat de València (UV), 46003 Valencia, Spain; esperanza.navarro@uv.es; 5 Department of Neurology, Hospital Clínico San Carlos, 28040 Madrid, Spain; vicmayordomo@gmail.com (V.G.-M.); mlcuadrado@med.ucm.es (M.L.C.); 6 Department of Medicine, School of Medicine, Universidad Complutense de Madrid, 28040 Madrid, Spain; 7 School of Health Sciences, Universidad Alfonso X el Sabio, 28691 Madrid, Spain; josari@uax.es; 8 Department of Psychology, Universidad Rey Juan Carlos (URJC), 28922 Madrid, Spain; margarita.cigaran@urjc.es; 9 Department of Public Health, Universidad Rey Juan Carlos (URJC), 28922 Madrid, Spain; valentin.hernandez@urjc.es; 10 Department of Medical Gastroenterology, Aalborg University Hospital, 9220 Aalborg, Denmark

We have read with great interest the comment by Ayuso García et al. [1] based on our article [2]. Ayuso García et al. [1] presented data confirming that females with long COVID reported lower health-related quality of life than men. These results complement those previously observed by our group [2] and those conclusions reported by a recent systematic review confirming that female sex is a risk factor for developing long COVID symptoms [3]. Current research supports that females, although they exhibit lower risk for severe acute infection and less mortality than males, suffer more post-COVID symptomatology at different aspects including physical, emotional, cognitive, and health-related quality of life [1,2,3]. Accordingly, this means that management of long COVID should be considered from a gender perspective since considering sex differences in diagnosis, prevention and treatment of diseases are fundamental steps towards precision medicine [4]. 

Several biological, emotional, and social gender differences should be considered and integrated when managing long COVID. Among the biological differences (e.g., genetics, hormones) two main factors would be related to long COVID. First, females have a greater expression of angiotensin-converting enzyme-2 (ACE2) and transmembrane protease serine 2 (TMPRSS2) receptors than males [5]. Since these receptors are the main cellular-to-cellular internal pathway of SARS-CoV-2, their higher expression could contribute to a higher viral load and a posterior persistence of the virus in females [5]. A second biological aspect would be a decreased innate immunological response (i.e., lower production of pro-inflammatory interleukin-6 (IL-6), after the acute phase of the infection in females) [6]. This reduced immunological response could lead to a rebound effect and a greater development of post-COVID symptoms [6]. Obviously, an effect of estrogens in these responses should be also taken into account [7]. Others have proposed that gender differences in brain development and functioning would explain biological differences between males and females [8,9].

Differences in emotional and cognitive behaviors could also explain gender differences in post-COVID symptoms. Females usually exhibit higher prevalence of mood disorders (e.g., depressive or anxiety levels and worse quality of sleep than males). This has been observed in particular during the pandemic/confinement since the COVID-19 outbreak’s surrounding factors such as isolation, fear of the infection, or uncertainty against a new and unexpected situation affected more females than males [10]. This is in agreement with findings suggesting that females are considerably more susceptible to secondary traumatization than male [11]. Since COVID-19 is being considered as post-traumatic stress disorder (PTSD), this stressful situation could induce a worse perception of health in females.

Finally, social factors intrinsic to gender should be also integrated into this equation. For instance, due to a higher fear perception, females exhibit different sanitary-related behaviors during this sanitary crisis (e.g., more frequent hand washing or less exposure to SARS-CoV-2 virus) than males [12]. However, the relevance of these factors with respect to the development of more severe post-COVID symptoms seems to be small, but should be considered for better understanding the different responses between males and females.

In conclusion, in agreement with Ayuso García et al. [1] and Fernández-de-las-Peñas et al. [2], long COVID symptoms (physical, cognitive and health-related) must be treated from a gender perspective. Future clinical trials investigating the effects of potential treatments for long COVID should integrate biological, emotional, and social differences between males and females.

## References

[1] Ayuso García B., Romay Lema E., Rabuñal Rey R. (2022). Health Perception among Female COVID-19 Patients. Comment on Fernández-de-las-Peñas et al. Female Sex Is a Risk Factor Associated with Long-Term Post-COVID Related-Symptoms but Not with COVID-19 Symptoms: The LONG-COVID-EXP-CM Multicenter Study. *J. Clin. Med.* 2022, *11*, 413.. J. Clin. Med..

[2] Fernández-de-las-Peñas C., Martín-Guerrero J.D., Pellicer-Valero O.J., Navarro-Pardo E., Gómez-Mayordomo V., Cuadrado M.L., Arias-Navalón J.A., Cigarán-Méndez M., Hernández-Barrera V., Arendt-Nielsen L. (2022). Female Sex Is a Risk Factor Associated with Long-Term Post-COVID Related-Symptoms but Not with COVID-19 Symptoms: The LONG-COVID-EXP-CM Multicenter Study. J. Clin. Med..

[3] Maglietta G., Diodati F., Puntoni M., Lazzarelli S., Marcomini B., Patrizi L., Caminiti C. (2022). Prognostic factors for Post-COVID-19 syndrome: A systematic review and meta-analysis. J. Clin. Med..

[4] Mauvais-Jarvis F., Merz N.B., Barnes P.J., Brinton R.D., Carrero J.-J., DeMeo D.L., De Vries G.J., Epperson C.N., Govindan R., Klein S.L. (2020). Sex and gender: Modifiers of health, disease, and medicine. Lancet.

[5] Bwire G.M. (2020). Coronavirus: Why men are more vulnerable to covid-19 than women?. SN Compr. Clin. Med..

[6] Anca P.S., Toth P.P., Kempler P., Rizzo M. (2021). Gender differences in the battle against COVID-19, Impact of genetics, comorbidities, inflammation and lifestyle on differences in outcomes. Int. J. Clin. Pract..

[7] Ortona E., Buonsenso D., Carfi A., Malorni W. (2021). Long Covid Kids study group. Long COVID: An estrogen-associated autoimmune disease?. Cell Death Discov..

[8] Slotnick S.D. (2021). Sex differences in the brain. Cogn. Neurosci..

[9] Wierenga L.M., Doucet G.E., Dima D., Agartz I., Aghajani M., Akudjedu T.N., Albajes-Eizagirre A., Alnæs D., Alpert K.I., Andreassen O.A. (2022). Greater male than female variability in regional brain structure across the lifespan. Hum. Brain Mapp..

[10] Metin A., Erbiçer E.S., Şen S., Çetinkaya A. (2022). Gender and COVID-19 related fear and anxiety: A meta-analysis. J. Affect. Disord..

[11] Baum N., Rahav G., Sharon M. (2014). Heightened susceptibility to secondary traumatization: A meta-analysis of gender differences. Am. J. Orthopsychiatry.

[12] Salari N., Hosseinian-Far A., Jalali R., Vaisi-Raygani A., Rasoulpoor S., Mohammadi M., Rasoulpoor S., Khaledi-Paveh B. (2020). Prevalence of stress, anxiety, depression among the general population during the COVID-19 pandemic: A systematic review and meta-analysis. Glob. Health.

